# Immune Response of Nile Tilapia ( *Oreochromis niloticus* ) Vaccinated With Diatom‐Based Oral Vaccines Against Piscine Francisellosis

**DOI:** 10.1111/jfd.14111

**Published:** 2025-03-01

**Authors:** Collin Meyer, Roshan P. Shrestha, Ruth Milston‐Clements, Sarah Gibson, Taylor I. Heckman, Zeinab Yazdi, Esteban Soto

**Affiliations:** ^1^ Department of Medicine and Epidemiology School of Veterinary Medicine, University of California Davis California USA; ^2^ PhycoVax LLC San Diego California USA; ^3^ Department of Microbiology, College of Science Oregon State University Corvallis Oregon USA; ^4^ Department of Wildlife, Fisheries and Aquaculture College of Forest Resources, Mississippi State University Starkville Mississippi USA; ^5^ Thad Cochran National Warmwater Aquaculture Center, Mississippi Agriculture and Forestry Experiment Station Mississippi State University Stoneville Mississippi USA

**Keywords:** cytokine, diatom, *Francisella orientalis*, immersion challenge, oral vaccine, tilapia

## Abstract

Piscine francisellosis is a highly infectious and economically significant disease caused by *Francisella orientalis* in tilapia (*Oreochromis* spp.). There are currently no approved treatments or commercial vaccines for this disease in cultured fish. Injectable vaccines using diatoms as antigen expression vectors have demonstrated efficacy in tilapia models; however, no oral vaccine trials have been performed. We hypothesised that fusion proteins consisting of *F. orientalis* IglC and flagellin expressed in *Thalassiosira pseudonana* diatoms will act as self‐adjuvanting antigen delivery systems to confer a protective immune response against *F. orientalis* in tilapia when administered as top‐coated feed. Different treatments were immunised and subsequently provided with one or two boosters prior to challenge. Fish were challenged with virulent *F. orientalis* via immersion thirty days post initial immunisation. Tilapia immune response was assessed 24 h post‐challenge by quantifying *il‐12, il‐10, ifn‐γ* and *tgf‐β* gene expression in gills and internal organs. Morbidity and mortality were monitored for 21 days after challenge and bacterial load was assessed in survivors. Findings indicate significant changes in the expression of *ifn‐γ* and *tgf‐β* in immunised fish, but similar mortality rates and bacterial load across all exposed groups.

## 1 Introduction

Tilapia (*Oreochromis* spp.) are the second most widely farmed fish worldwide, with an estimated market value of $12 billion USD (Peñarubia et al. 2022). However, infectious diseases continually plague the industry, resulting in significant financial losses (Food and Agriculture Organization of the United Nations 2024; Kobayashi et al. 2015). Piscine francisellosis is a leading disease in tilapia aquaculture, caused by the gram‐negative, facultative intracellular bacterium *Francisella orientalis*. It presents as a systemic granulomatous disease resulting in mortalities of up to 95%, delayed growth of survivors, and decreased profitability of production fish (Birkbeck, Feist and Verner‐Jeffreys 2011). The pathogen has a high infection rate, multiple transmission routes, and can persist in the environment in a viable state for > 60 days (Duodu and Colquhoun 2010; Mauel et al. 2003; Soto et al. 2011). Despite the importance of piscine francisellosis, there is a lack of prophylactic and therapeutic methods available.

Advances in biotechnology research have shown that diatoms, a form of microalgae, can undergo transformation to express recombinant bacterial proteins (Davis et al. 2017; Specht and Mayfield 2014). This novel technology has been used to develop innovative vaccine candidates that are safer than live‐attenuated or DNA vaccines because they cannot invade the host's genome or replicate in the environment or host. Recently, *Thalassiosira pseudonana* diatoms expressing the immunodominant *F. orientalis* intracellular growth loci C protein (IglC) were evaluated with either a nanoparticle or a Montanide (Seppic, France) adjuvant (Shahin et al. 2020). Both forms of the vaccine resulted in significant immunostimulation and protection for tilapia in laboratory‐controlled challenges when injected intracoelomically, indicating the effectiveness of the IglC fusion protein as an antigen (Shahin et al. 2020). However, parenteral vaccines are less attractive for production fish as they tend to result in poor mucosal immune stimulation, require potentially stressful handling of the fish, are time and labour consuming, and can only be administered to larger‐sized fish (Shahin et al. 2020).

In contrast, oral vaccines are less stressful and more practical. They eliminate the need to handle thousands of individuals at a time and have been shown to improve mucosal immune response, an important component of the fish defence system (Hoare et al. 2021; Parra 2015; Specht and Mayfield 2014). Propitiously, diatoms are already commonly used as a nutritional supplement in aquafarms, which offers the possibility of using them to immunise fish via their feed. In order to generate an immune response, oral vaccines must deliver their antigen through the digestive system and into circulation. Transgenic diatoms have been shown to be capable of this in vitro when exposed to the bile salts and acidic conditions found in fish digestive tracts, further supporting their proposed use as an oral antigen delivery system (Kiataramgul et al. 2020).

This study aimed to leverage the advantages of orally administered diatom‐based vaccines to protect against highly fatal francisellosis in tilapia. We developed transgenic *T. pseudonana* diatoms co‐expressing *F. orientalis* IglC as an immunodominant antigen, bacterial flagellin as an adjuvant and green fluorescent protein (GFP) to aid in cell selection during diatom transformation (Doan et al. 2020; Shahin et al. 2018, 2020; Wangkahart, Secombes, and Wang 2019). The potential vaccine was then investigated using a Nile Tilapia ( *Oreochromis niloticus* ) model of infection with *F. orientalis*, evaluating morbidity, mortality, bacterial load and cytokine expression in systemic and mucosal sites post‐infection.

## 2 Materials and Methods

### 2.1 Cloning and Expression of the Antigen‐Adjuvant Fusion Protein in Diatoms

The genes encoding *Salmonella typhimurium* flagellin *fliC* (adjuvant) and *F. orientalis* pathogenicity island protein *iglC* (antigen) were codon‐optimised for the diatom *T. pseudonana* using the Kazusa Codon Usage Database (Nakamura, Gojobori, and Ikemura 2000). The optimised genes were synthesised as double‐stranded DNA using gBLocks Gene Fragments (Integrated DNA Technologies, Coralville, Iowa, USA) and cloned into a pDONR plasmid via Gateway BP cloning (Invitrogen, Carlsbad, California, USA). This plasmid was then cloned into a diatom destination vector as previously described (Shrestha and Hildebrand 2015). The resulting diatom expression plasmid vector contained 5′‐*fliC*, *iglC* and 3′‐*gfp*, all under the transcriptional control of the fucoxanthin chlorophyll a/c‐binding gene (*fcp*) promoter and terminator. Control plasmid vectors IglC‐eGFP and flagellin‐eGFP were similarly constructed.

The transformation vector (flagellin‐IglC‐eGFP) was introduced into the centric diatom *T. pseudonana* cells using the gene gun (particle bombardment) method, leading to random integration of the transgene into the nuclear genome (Apt 1996). Control plasmid vectors expressing IglC‐eGFP and flagellin‐eGFP were also transformed into *T. pseudonana* cells to compare the antigen and vector backbone. Each transformation vector was co‐transformed with a vector expressing the *nat1* gene, which provides resistance to the antibiotic nourseothricin (Poulsen, Chesley and Kroger 2006).

Following the transformation, diatom cells were cultured in artificial seawater (ASW) medium with 100 μg/mL nourseothricin. The cells underwent two rounds of sorting via flow cytometry to isolate those with high levels of eGFP‐tagged recombinant protein. The final sorted cells were plated on ASW‐agar plates, and clones with the highest GFP expression were selected. Green fluorescent protein quantification was performed by measuring GFP fluorescence according to the protocol outlined in Technical Note 170 by DeNovix (Jones 2020). The measurements were carried out using ThermoFisher's Qubit 4 fluorometer. The excitation wavelength was 470 nm, and fluorescence emission was recorded in the 510–580 nm range. These selected clones were then grown in 125‐mL flasks and later expanded in a PBR 1250‐L photobioreactor (Industrial Plankton, Victoria, British Columbia, Canada). The eGFP‐tagged recombinant fusion protein was continuously expressed under the control of a strong constitutive Fcp promoter. IglC content was quantified using the fusion partner GFP as a proxy, and the calculation was based on a molecular mass ratio of IglC to GFP of 0.83:1. Cells were harvested by centrifugation, freeze‐dried, vacuum‐packed and stored at −20°C (Ahmed et al. 2015). Wild‐type diatoms were harvested and stored in the same manner.

### 2.2 Fish and Rearing Conditions

The experimental protocol was approved by the Institutional Animal Care and Use Committee of Oregon State University. Nile Tilapia fingerlings (*N* = 1240) were acquired from Aquasafra Inc. (Bradenton, Florida, USA) at an average weight of 0.5 g and transported to the John L Fryer Aquatic Animal Health Laboratory, Oregon State University, Corvallis, USA. Tilapia were allowed to acclimate for 2 weeks in 100‐L flow‐through (1 L/min) indoor tanks with UV‐sterilised and degassed well water at 25°C. Water temperature and flow rate in each tank were monitored daily. No other water quality parameters were evaluated during the study. Annual water quality analysis of the source well water was completed by National Testing Laboratories, Cleveland, Ohio, USA and is reported in Table S1 of the supplemental materials. Fish were fed a starter commercial tilapia feed (Bio‐Oregon, Longview, Oregon, USA) at a rate of 2% body weight per day.

### 2.3 Fish Immunisation

Tilapia were arbitrarily divided into 17 groups (*n* = 73 fish per treatment) based on vaccine type, number of administrations and challenge assignment as described in Table 1, and shown visually in Data S1. Vaccine feed was prepared by mixing a 1:1:1 ratio (*w*/*w*/*v*) of commercial feed, respective diatom treatment, and Wild Caught OMEGA‐3 fish oil. After the acclimation period, the fish were fasted for 24 h, then provided their experimental diet at a rate of 1% body weight twice a day for two consecutive days at the start of week one. Using the described dosing regime, the fish receiving the full fusion flagellin‐IglC diatom treatment were exposed to a dose of 36.8 μg/g fish biomass. Assigned groups received their first booster using the same procedure at the start of week two, and those receiving a second booster repeated the procedure again in week four. A visual timeline of the vaccination protocol is provided in Data S1. During the third week of the immunisation period, each group was randomly subdivided (*n* = 18) into 4 replicate 25‐L flow‐through (1 L/min) indoor tanks at 25°C in anticipation of the upcoming bacterial challenge. Water source and chemistry parameters were the same as in the previous 100‐L tanks. Flow rate was equivalent in all tanks and measured manually with graduated cylinders.

**TABLE 1:** Experimental groupings of Nile Tilapia challenged with *Francisella orientalis* or a placebo. Fish were divided into 17 groups (A‐Q, *n* = 73 each), with each group receiving a variation of a recombinant oral vaccine. The dosing regimens consisted of an initial dose only, single‐booster (given 1 week after initial dose), and double‐booster (given 1 week and 3 weeks after initial dose) protocols.

Group	Challenge assignment	Vaccine type	Dose
A	Placebo	Commercial feed only	—
B	Placebo	Commercial feed with eGFP‐flagellin‐IglC	Initial only
C	Placebo	Commercial feed with eGFP‐flagellin‐IglC	Initial + 1 booster
D	Placebo	Commercial feed with eGFP‐flagellin‐IglC	Initial + 2 boosters
E	Challenged	Commercial feed only	—
F	Challenged	Commercial feed with wild‐type diatoms	Initial only
G	Challenged	Commercial feed with wild‐type diatoms	Initial + 1 booster
H	Challenged	Commercial feed with wild‐type diatoms	Initial + 2 boosters
I	Challenged	Commercial feed with eGFP‐IglC	Initial only
J	Challenged	Commercial feed with eGFP‐IglC	Initial + 1 booster
K	Challenged	Commercial feed with eGFP‐IglC	Initial + 2 boosters
L	Challenged	Commercial feed with eGFP‐flagellin	Initial only
M	Challenged	Commercial feed with eGFP‐flagellin	Initial + 1 booster
N	Challenged	Commercial feed with eGFP‐flagellin	Initial + 2 boosters
O	Challenged	Commercial feed with eGFP‐flagellin‐IglC	Initial only
P	Challenged	Commercial feed with eGFP‐flagellin‐IglC	Initial + 1 booster
Q	Challenged	Commercial feed with eGFP‐flagellin‐IglC	Initial + 2 boosters

### 2.4 Bacteria and Culture Conditions

*Francisella orientalis* LADL‐07‐285A, previously isolated from diseased Nile Tilapia, was cultured on Modified Thayer‐Martin (MTM, BD BBL, Franklin Lakes, New Jersey, USA) for 72 h at 28°C (Soto et al. 2009). Colonies were then transferred to Modified Muller Hinton broth (Sigma‐Aldrich, St. Louis, Missouri, USA) and incubated at 28°C and 160 rpm for 18 h (Soto et al. 2009). The number of colony‐forming units (CFU) per mL was estimated using the drop plate method on MTM, incubated for 72 h at 28°C.

### 2.5 Fish Challenge and Sampling

Thirty days following initial immunisation, treatments E‐Q (Table 1) were challenged with 1.6 × 10^5^ CFU *F. orientalis*/mL tank water for 5 h at 25°C via immersion. Unchallenged control groups A–D (Table 1) were similarly exposed to sterile media during this time. Water flow was restricted for the duration of the immersion to create a static (0 L/min flow‐through) environment, then restored to 1 L/min flow‐through in all tanks after completion of the 5‐h challenge. Tanks were oxygenated with individual air stones in each tank.

Twenty‐four hours after immersion, 2 fish per replicate tank (*n* = 8 per treatment group) were euthanised via overdose of sodium bicarbonate buffered tricaine methanesulfonate (MS‐222) (Syndel, Ferndale, Washington, USA) at 500 mg/L. Gills and internal organs from each fish were aseptically harvested and saved in separate sterile 1.5 mL Eppendorf tubes containing 500 μL of RNAlater (Invitrogen, Carlsbad, CA, USA). The internal organs were harvested collectively due to the small size (< 1 g) of the fish.

Morbidity and mortality were monitored for 21 days post‐challenge (dpc). The first five mortalities per treatment were collected immediately once observed and saved at −20°C for testing. The presence of pathogen DNA was later confirmed and quantified via qPCR (Soto et al. 2010) as described below.

At the completion of the trial, the relative percent survival was calculated for each treatment, and all remaining fish were euthanized using an overdose of buffered MS‐222 (Syndel, Ferndale, Washington, USA) at 500 mg/L. Ten arbitrarily selected surviving fish per treatment were saved at −20°C, and the presence of the pathogen DNA was confirmed and quantified via qPCR (Soto et al. 2010). A visual timeline of these methods has been provided in Data S1.

### 2.6 Transcript Quantification of Immune‐Related Genes

Total RNA from the gills and internal organs collected at 24 h post‐challenge was extracted using the RNeasy plus MiniKit (Qiagen, Hilden, Germany) following the manufacturer's instructions. Complementary DNA (cDNA) was then synthesised from approximately 1 μL RNA using the QuantiTect Reverse Transcription kit (Qiagen, Hilden, Germany) following manufacturer's instructions. The resulting product was then diluted to a total volume of 100 μL. Quantitative PCR was performed on the cDNA product in duplicate using a SYBR Green kit (ThermoFisher, Waltham, Massachusetts, USA) following the manufacturers' instructions, utilising the primers listed in Table 2. Resulting threshold cycle (Ct) values were then averaged and transformed with 2−^∆∆Ct^, using *actb* and *ubce* as reference genes to determine relative expression (Yang et al. 2013).

**TABLE 2:** Primers used in this study. ‘‐F’ or ‘‐S1’ in the primer name indicates a forward primer, ‘‐R’ or ‘A1’ indicates a reverse primer.

Gene	Primer name	NCBI or published reference	Primer sequence (5′‐3′)
β‐Actin (*actb*)	Til‐βactin‐F Til‐βactin‐R	Wang et al. 2013	AACAACCACACACCACACATTTC TGTCTCCTTCATCGTTCCAGTTT
Ubiquitin‐conjugating enzyme (*ubce*)	UBCE‐S1 UBCE‐A1	Yang et al. 2013	CTCTCAAATCAATGCCACTTCC CCCTGGTGGAGGTTCCTTGT
Interferon gamma (*ifn‐γ*)	Til‐IFNG‐F Til‐IFNG‐R	GL831195.2	ACCACATCGTTCAGAGCAAAG CCTCTGGATCTTGATTTCGGG
Interleukin 12 (*il‐12*)	Til‐IL12‐F Til‐IL12‐R	XM_025896926.1	TGGACAGGCAATGGATACAC AGGGTGAGAGGTCGTGATAC
Transforming growth factor β (*tgf‐β*)	Til‐TGFb‐F Til‐TGFb‐R	XM_025897821.1	ACCATGTCCATCCCTAAAACC CCACTTCCATCCTAGATCTTTCC
Interleukin 10 (*il‐10*)	Til‐Il10‐F Til‐Il10‐R	GL831146.2	TGGAATTTTACCTGGACACGG GGAGAAGTATTTCCTACACTGGG
Intracellular growth loci C (*iglC*)	Fo‐iglC‐F Fo‐iglC‐R Fo‐iglC‐Probe	Soto et al. 2010	GGGCGTATCTAAGGATGGTATGAG AGCACAGCATACAGGCAAGCTA FAM‐ATCTATTGATGGGCTCACAACTTCACAA‐BHQ‐1

### 2.7 Quantification of Bacterial DNA Load in Survivor Fish

Internal organs from each of the collected mortalities and the euthanised survivors were aseptically harvested and saved in sterile 1.5 mL Eppendorf tubes. DNA was extracted from these samples using the DNeasy Blood & Tissue Kit (Qiagen, Hilden, Germany) following the manufacturer's instructions. DNA was diluted to 20 ng/μL before qPCR evaluation using the primer‐probe combination listed in Table 2. Resulting Ct values were then averaged and transformed to genome equivalents using a standard curve to estimate bacterial load (Soto et al. 2010).

### 2.8 Statistical Analysis

Figures and statistical analyses were generated using GraphPad Prism (v10.0.0). Survival curve analysis used the Kaplan–Meier method followed by a Log‐rank (Mantel Cox) test for significance and was assessed using the Bonferroni correction test at *α* = 0.05. Mortality rate, bacterial load and cytokine expression analyses used a one‐way ANOVA with a Tukey post hoc test. All tests were considered to be significant at *p* < 0.05.

## 3 Results

### 3.1 Morbidity and Mortality

**FIGURE 1:**
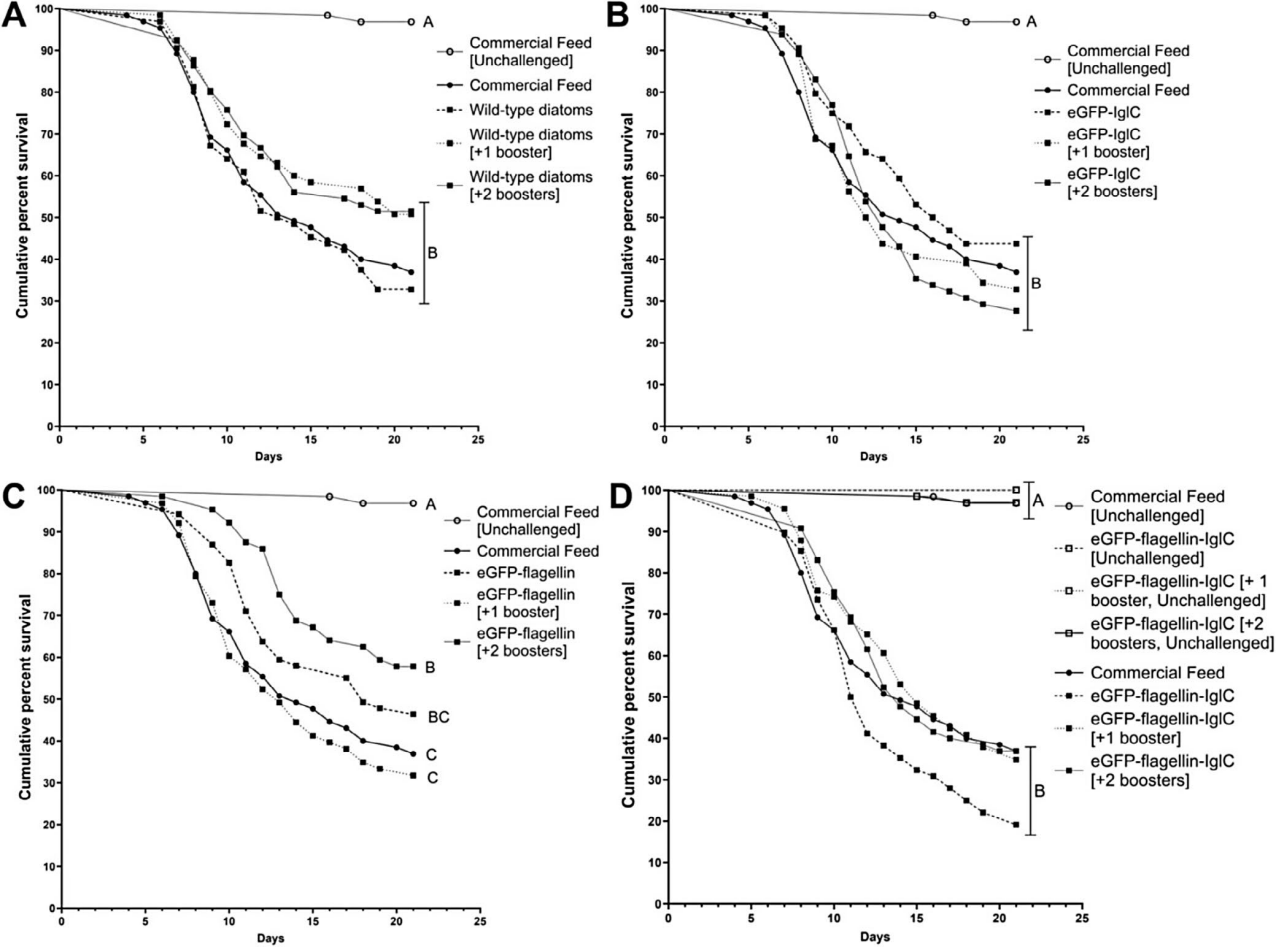
Kaplan–Meier survival curves of tilapia fingerlings from day 0 to day 21 post immersion challenge with *Francisella orientalis*. Each curve represents the average survival of 4 parallel tanks holding *n* = 16 fish. Groups that do not share letters are significantly different as determined by Log‐rank (Mantel Cox) test with Bonferroni correction (*α* = 0.05). (A) Wild‐type diatoms, (B) flagellin‐expressing diatoms, (C) IglC‐expressing diatoms and (D) flagellin‐IglC‐expressing diatoms, including both challenged and unchallenged groups.

**FIGURE 2:**
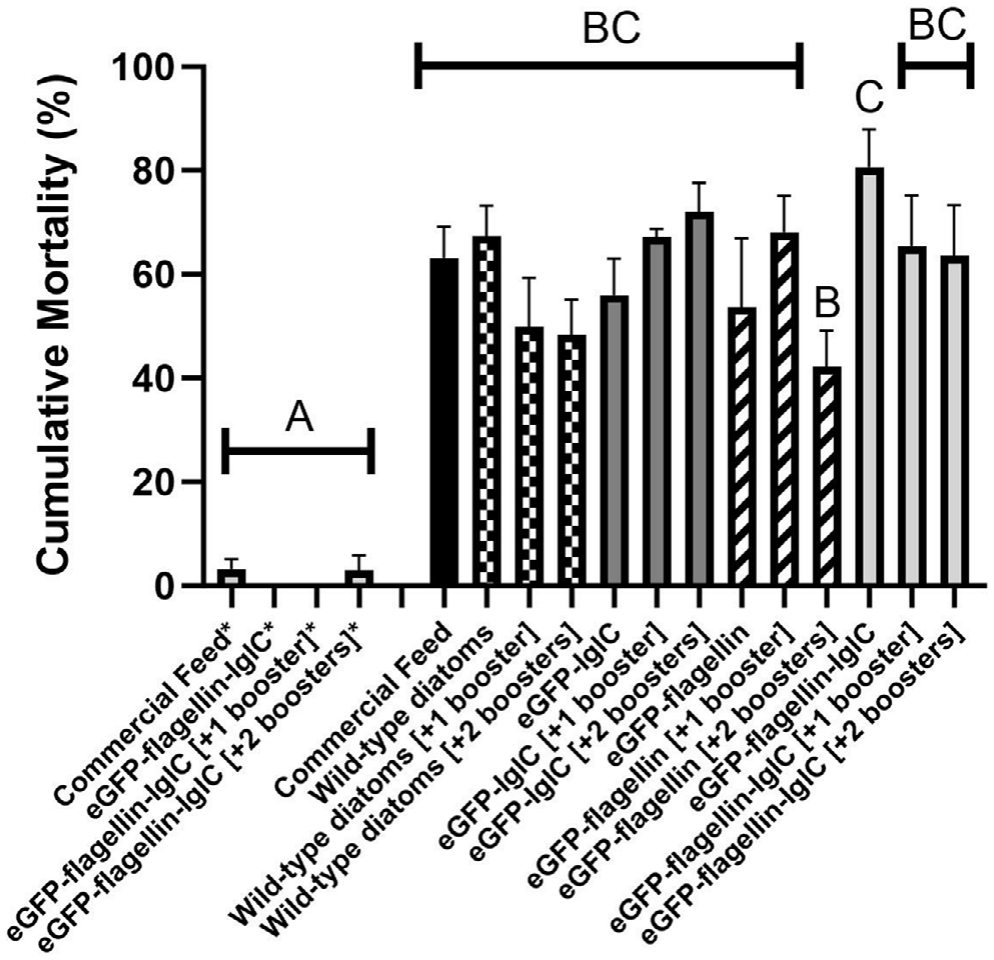
Cumulative mortality rate of tilapia fingerlings 21 days post challenge with 1.6 × 10^5^ CFU *Francisella orientalis*/mL tank water for 5 h at 25°C. Each column represents the mean with standard error of 4 replicate tanks (*n* = 16 fish each). Columns with asterisks (*) were unchallenged controls. Groups that do not share letters are significantly different (*p* < 0.05) as determined by one‐way ANOVA with post hoc Tukey Test.

Fish challenged with *F. orientalis* began to show clinical signs and mortalities around 7 ± 3 dpc, regardless of the experimental group (Figure 1). Clinical signs observed in moribund fish were consistent with piscine francisellosis, including anorexia, lethargy and organomegaly. At the completion of the trial, survival curves (Figure 1) and cumulative mortality rates (Figure 2) for the challenged treatment groups were significantly different from the unchallenged treatments. Only the group that received the flagellin treatment with two boosters had a significantly improved (*p* < 0.01) survival curve when compared to the positive control (Figure 1C). All other challenged experimental groups presented similar mortality rates (42%–80%) to the sham‐vaccinated positive control (63% ± 6.1%) (Figures 1 and 2). The unchallenged experimental groups that received the full fusion flagellin‐IglC diatom treatment had only 0%–3% mortality, the same as was observed in the negative control group (Figure 1D, Figure 2).

**FIGURE 3:**
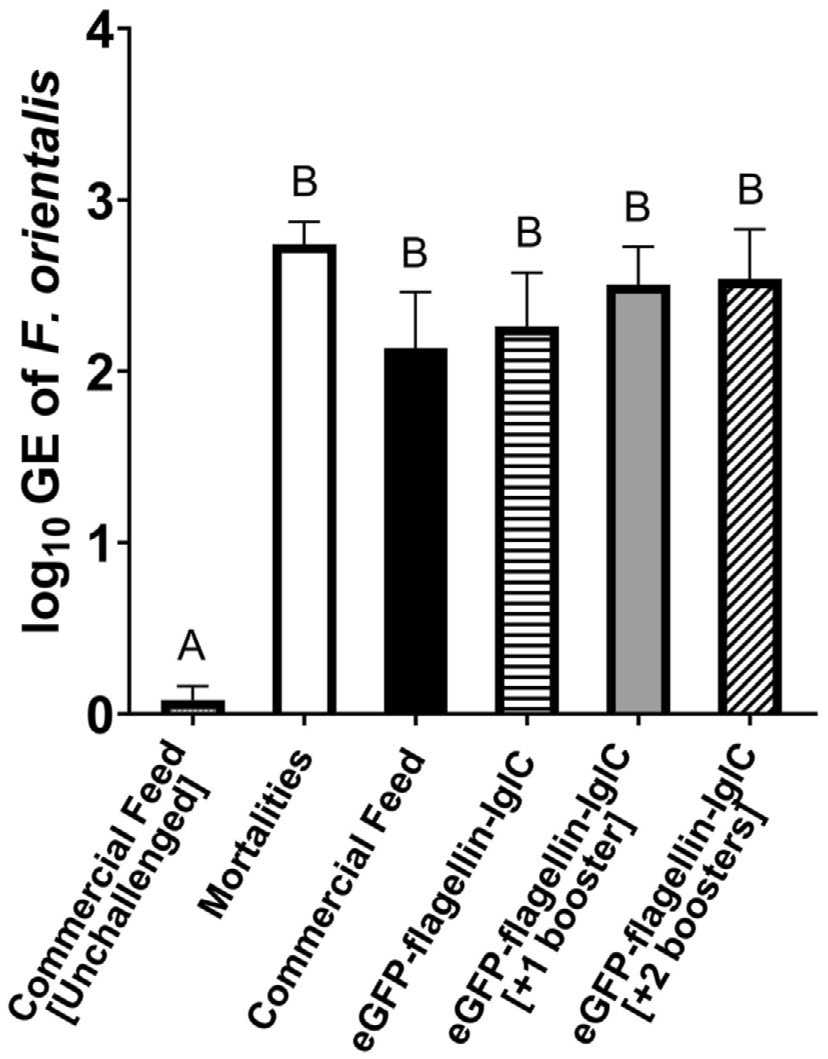
*Francisella orientalis* genome equivalent (GE) quantification in internal organs of tilapia fingerlings by qPCR. Treatments (*n* = 10/group) sampled from survivors at 21 days post challenge with 1.6 × 10^5^ CFU *F. orientalis*/mL tank water for 5 h at 25°C. Mortalities (*n* = 20) across groups were sampled at the time of death. Columns represent mean with standard error. Different letters are significantly different (*p* < 0.05) as determined by one‐way ANOVA with post hoc Tukey Test.

*Francisella orientalis iglC* was detected in the internal organs of all mortalities from both the challenged fusion protein treatment groups and the positive control group (Figure 3). The mortalities had no significant difference in *F. orientalis* genome equivalents when compared to the euthanized survivors from the same groups. *Francisella orientalis iglC* was also detected in one of the ten negative control fish at a low concentration (0.81 log_10_ genome equivalents of *F. orientalis* , Figure 3).

### 3.2 Immunoassay

**FIGURE 4:**
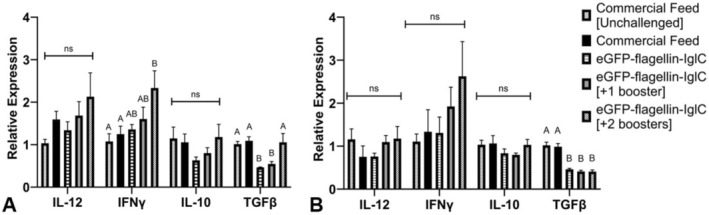
Relative gene expression of interleukin 12, interferon gamma, interleukin 10 and transforming growth factor beta in (A) gills and (B) internal organs of *n* = 8/group tilapia fingerlings 24 h post challenge with 1.6 × 10^5^ CFU *Francisella orientalis*/mL tank water for 5 h at 25°C. Columns represent mean with standard error. Gene expression was normalised against the expression of the average of *actb* and *ubce* reference genes. Different letters within each gene are significantly different (*p* < 0.05) as determined by one‐way ANOVA with post hoc Tukey Test.

Relative gene expression was tested at 24 h post‐infection in the positive and negative controls, as well as in the challenged fusion protein groups. Relative gene expression of *il‐12* and *il‐10* was similar in the gills and internal organs of the experimental groups when compared to their respective controls (Figure 4). In the gills alone, *ifn‐γ* was significantly upregulated in the fusion protein group that received two boosters when compared to the control groups. Also in the gills, *tgf‐β* expression was significantly decreased in the immunised fish, except for the group that received two boosters of the fusion protein treatment (Figure 4A). In the internal organs, all fusion protein treatment groups showed a significant decrease in *tgf‐β* expression compared to the controls (Figure 4B).

## 4 Discussion

While oral vaccines simplify the process of immunising fish, they are more difficult to standardise dosage for in a manner that can consistently stimulate an efficacious immune response through mucosal routes (Mutoloki 2015). In the case of the flagellin‐IglC fusion proteins expressed in diatoms, vaccination by oral administration produced no discernible decreases in mortality from *F. orientalis* infection in Nile Tilapia fingerlings (Figure 1, Figure 2). Mortalities observed in this study were confirmed to have been infected with *F. orientalis* , presenting substantial quantities of *iglC* DNA. Sampled survivors also had unexpectedly similar levels of *iglC* DNA identified in their internal organs as the mortalities (Figure 3). This may be due, in part, to a smaller sample size of surviving fish compared to mortalities, but more likely it is a result of the disease still progressing through the remaining population of fish. Figure 1 shows how, although the mortality rate had slowed at 21 dpc, it had not yet plateaued, indicating that infection and death were still occurring at the end of the challenge. One of the negative control fish also had detectable quantities of *iglC* (Figure 3). Due to the strict organ harvesting protocols, low quantity of DNA recorded and lack of infection in tankmates, this result is most likely a cross‐contamination from when the survivor fish was harvested and transported as opposed to a true infection. All unchallenged fish lacked pathologic signs of francisellosis and were otherwise negative for the presence of *iglC*.

Interestingly, the group that received the flagellin treatment with two boosters had the lowest mortality rate (Figure 2) and a significantly improved survival curve (Figure 1C). Flagellin is known to act as a potent bacterial immunostimulant for a wide variety of immune cells expressing the relevant toll‐like receptor TLR5 (Doan et al. 2020; Wangkahart, Secombes, and Wang 2019). As such, it was originally included in the fusion protein to remove the need for an external adjuvant. Its independent effect on improving tilapia survival may be related to the emerging concept of trained immunity (Byrne, Loving, and McGill 2020).

Innate immune cells, in particular monocytes, have the capacity to undergo epigenetic changes following a primary stimulus (Byrne, Loving, and McGill 2020; Petit et al. 2019; Petit and Wiegertjes 2016). These changes may occur in progenitor cells and can lead to either a sensitised (trained) or desensitised (tolerant) state in descendent cells faced with a secondary stimulus many days to months later. Furthermore, this altered reactivity is generally non‐specific, meaning that a primary pathogen‐associated molecular pattern (PAMP) may increase sensitivity to multiple, different secondary pathogenic patterns if they interact with similar receptors (Byrne, Loving, and McGill 2020; Petit et al. 2019; Petit and Wiegertjes 2016). This effect is highly context dependent, where both primary and secondary stimuli sources, specific molecular orientations, concentrations, species of training and administration routes can all alter or eliminate the trained effect (Ifrim et al. 2014; Byrne, Loving, and McGill 2020; Petit et al. 2019; Petit and Wiegertjes 2016). Even then, if the primary stimulus is successful in the initiation of the trained immunity response, it may not always be beneficial. A sensitive immune system has the potential to instead cause increased pathology and impaired production qualities rather than a specific protective effect (Byrne, Loving, and McGill 2020). Very young animals, like the fingerlings used in this study, are more reliant on innate immune responses, and fish in particular have a highly diverse and active innate immune system in comparison to mammals; both of which indicate that the animals used in our study had a greater potential to exhibit aspects of immune training (Byrne, Loving, and McGill 2020; Petit and Wiegertjes 2016).

Flagellin and TLR‐activation have been demonstrated to induce both trained and tolerant macrophage epigenetic alterations in humans, albeit in an inconsistent, inversely dose‐dependent manner (Ifrim et al. 2014; Byrne, Loving, and McGill 2020; Petit et al. 2019). More well studied across humans, rodents and fish is the trained immunity observed by β‐glucans administration, which is the primary form of glucose storage and a component of cell walls in diatoms (Byrne, Loving, and McGill 2020; Petit et al. 2019; Petit and Wiegertjes 2016; Ahmad, Tiwari, and Srivastava 2020). In a purified form, β‐glucans have been a common aquaculture supplement since the 1990's and studied in fish including salmonids, carp and Nile Tilapia (Byrne, Loving, and McGill 2020; Petit et al. 2019; Petit and Wiegertjes 2016). While there remains a lack of knowledge regarding the mechanism in aquatic species, increased cytokine expression, phagocytic capacity, oxidative burst and lysozyme activity have all been noted in supplemented fish (Byrne, Loving, and McGill 2020; Petit et al. 2019; Petit and Wiegertjes 2016). In addition, orally administered β‐glucans have been shown to increase protection against multiple bacterial pathogens in fish (Petit and Wiegertjes 2016). This made the use of diatoms an ideal vector candidate for this study as the naturally produced β‐glucan could potentially act as an additional adjuvant and induce trained immunity. Protection was only observed in the flagellin‐vaccinated group, which received two booster administrations, with the last booster administered 7 days before the infectious challenge (Figure 1). Previous studies indicate that innate immune training effects last for thirty days or less, potentially explaining why un‐ or singly‐boosted groups did not show significant improvements (Byrne, Loving, and McGill 2020). What this hypothesis does not explain is why the combination IglC‐flagellin double‐boosted group did not also demonstrate benefits to fish survival, even though it too was bolstered 7 days prior to the infectious challenge (Figure 1).

Immune analysis (Figure 3, Figure 4) was limited to the controls and flagellin‐IglC treatment groups to focus on any effects that the fusion protein specifically incurred. *ifn‐γ* is a critical proinflammatory cytokine used to activate macrophages and major histocompatibility complex (MHC) class II cells (Yoshimura, Wakabayashi, and Mori 2010). Transcripts of *ifn‐γ* were elevated in the gills of fish that received the fusion protein treatment within 24 h post‐infection, indicating mucosal immunostimulation occurred. There was a distinct visual trend of increasing expression with increased administrations of the treatment, though statistical significance was observed only at the highest cumulative dose (Figure 4A). Under the immersion challenges used in this study, the gills would be one of the first organs in contact with the pathogen and therefore one of the first sites of immunostimulation. The same *ifn‐γ* response was not observed in the internal organs, which contain some of the main target tissues (secondary lymphoid organs) of this pathogen (Figure 4B). Given the small (< 1 g) size of the fish, individual organs were not able to be separated for testing, effectively creating a mixed sample that may have diluted tissue‐specific patterns. This is evident when observing the large standard errors for *ifn‐γ* expression in the internal organ sample, once again presenting a non‐significant yet clear visual trend.

*tgf‐β* is a pleiotropic cytokine which primarily inhibits macrophage production of pro‐inflammatory signals while stimulating the production of anti‐inflammatory signals. This cytokine also plays an important role in controlling T‐helper cell (Th) activation. *tgf‐β* prevents Th1 and Th2 differentiation and simultaneously creates regulatory T‐cells (Tregs) to establish tolerance (Castro‐Sánchez and Martín‐Villa 2013). However, it has been shown that naïve Th cells exposed to both *tgf‐β* and the local inflammatory cytokine IL‐6 will instead promote the generation of pro‐inflammatory Th17 cells (Yoshimura, Wakabayashi, and Mori 2010). Th1 cells are best suited to combat intracellular pathogens such as *F. orientalis* but are under counter‐regulatory control from the Th17 and Tregs generated by *tgf‐β* (Yoshimura, Wakabayashi, and Mori 2010). Our data demonstrated a reduction in acute *tgf‐β* expression in both the gills and internal organs following the challenge (Figure 4), which indicates the vaccinated fish may mount a more efficacious immune response when compared to non‐vaccinated fish.

As noted, there were multiple non‐significant but visually evident trends present in the cytokine data (Figure 4). In addition to substantiating evidence for increased *ifn‐γ* expression, proinflammatory *il‐12* and anti‐inflammatory *il‐10* should be investigated in the future with a larger sample size and more tissue‐specific sampling to verify trends without statistical backing. Additionally, as fry were used in the study (< 1 g in size), future studies evaluating the vaccine in older fish may result in stronger immune responses. Future research evaluating different dosing regimens, different adjuvants/immunostimulants and other antigen combinations that could convey a clinically relevant protective immune effect is warranted.

## 5 Conclusion

We demonstrated that the generated recombinant eGFP‐flagellin‐IglC fusion proteins expressed in diatoms are safe to consume by Nile Tilapia fingerlings and can induce a mucosal and systemic immune response. However, the evaluated treatments in fish < 1 g in size were ineffective in reducing mortality or infection by *F. orientalis* . Although the novel delivery method shows promise, further research is needed to provide a safe and effective vaccine against this disease.

## Author Contributions

**Collin Meyer:** investigation, writing – original draft, writing – review and editing, formal analysis. **Roshan P. Shrestha:** conceptualization, investigation, funding acquisition, writing – review and editing, methodology, software, data curation, project administration. **Ruth Milston‐Clements:** investigation, formal analysis, project administration, supervision, resources, writing – review and editing. **Sarah Gibson:** investigation, formal analysis, writing – review and editing. **Taylor I. Heckman:** conceptualization, investigation, writing – review and editing, validation, methodology, supervision, formal analysis. **Zeinab Yazdi:** investigation, formal analysis, writing – review and editing. **Esteban Soto:** conceptualization, investigation, funding acquisition, writing – review and editing, project administration, supervision.

## Conflicts of Interest

The authors declare no conflicts of interest.

## Supporting information


Data S1.

## Data Availability

The data that support the findings of this study are available from the corresponding author upon reasonable request.
