# Supplementary material for: Immune Response of Nile Tilapia ( Oreochromis niloticus ) Vaccinated With Diatom‐Based Oral Vaccines Against Piscine Francisellosis
Source: J Fish Dis. 2025 Mar 1;48(7):e14111. doi: 10.1111/jfd.14111 (PMC12152294; doi:10.1111/jfd.14111)
Supplement: Supplementary file 1 — Data S1. [file JFD-48-e14111-s001.docx]

**Supplemental Material**


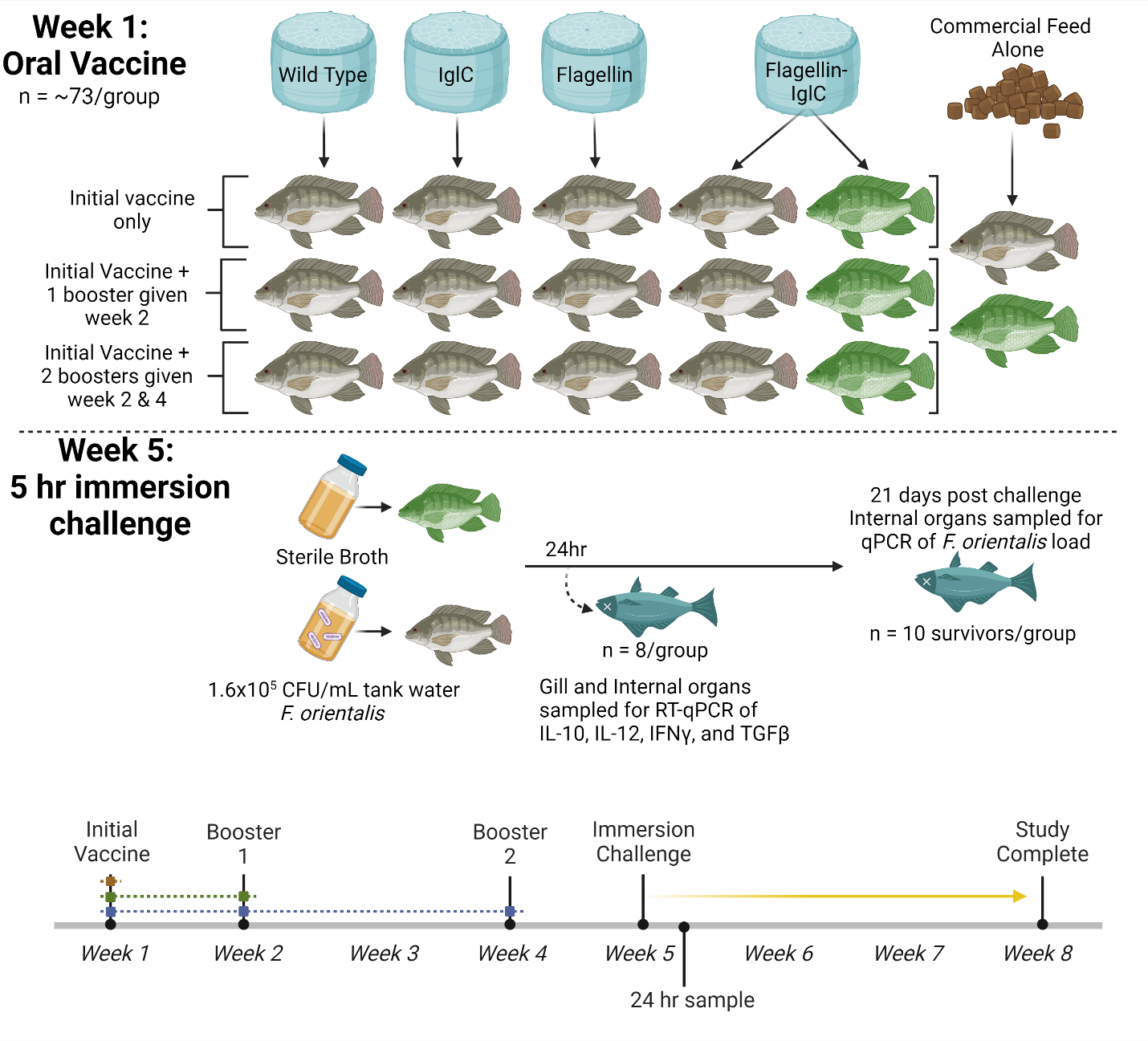


**Graphical Methods**: Experimental design depicting N = 1240 Nile tilapia fingerlings split amongst 17 treatment groups (n = 73). Each group received a combination of a diet and dosage regimen. Experimental fish were challenged or sham-challenged on week 5 after the initial vaccine with 1.6x10^5^ CFU *Francisella orientalis* / mL tank water for 5 hours at 25°C. 24 hours later, 8 fish per group were harvested to assess cytokine expression. Fish mortalities were monitored for 21 days post-challenge then 10 surviving fish per group were harvested to assess *F. orientalis* load. Image created with BioRender.

**Table 3:** Annual water parameter analysis of the John L Fryer Aquatic Animal Health Laboratory, Oregon State University, Corvallis, USA.

| Year | pH | Nitrite | Nitrate | Total alkalinity (as CaCO_3_) | Hardness |
| --- | --- | --- | --- | --- | --- |
| 2022 | 6.6 | <0.5 mg/L | 9.6 mg/L | 90 mg/L | 120 mg/L |
| 2021 | 6.6 | <0.5 mg/L | 9.0 mg/L | 80 mg/L | 130 mg/L |
| 2019 | 6.7 | <0.5 mg/L | 9.2 mg/L | 82 mg/L | 130 mg/L |
| 2018 | 6.7 | <0.5 mg/L | 8.8 mg/L | 84 mg/L | 130 mg/L |
